# Molecular dissection of tendon development and healing: Insights into tenogenic phenotypes and functions

**DOI:** 10.1016/j.jbc.2025.108353

**Published:** 2025-02-25

**Authors:** Takao Sakai, Ken Kumagai

**Affiliations:** 1 Department of Diagnostic Pathology, School of Medicine, Fujita Health University, Toyoake, Aichi, Japan; 2 Department of Orthopaedic Surgery, School of Medicine, Yokohama City University, Yokohama, Japan

**Keywords:** collagen, extracellular matrix, scleraxis, mechanical loading, mohawk, tenocytes, TGF-β

## Abstract

Tendon is a dense connective tissue that transmits contraction forces from skeletal muscles to bones. Adult tendon injury is a significant clinical problem because it occurs frequently with a high recurrence rate, and damaged tendon is rarely restored to full function. The main barrier to improving recovery outcomes is our incomplete understanding of the molecular mechanisms underlying the biological alterations following tendon injury *in vivo*. In this review, we specifically highlight the cellular dynamism of fibrotic tendon wound healing and the roles of mechanical loading. In particular, we document how tendon stem/progenitor cells expressing the tendon-specific transcription factor Scleraxis (Scx) play a role in fibrotic tendon wound healing, and describe novel experimental systems such as lineage cell tracing and single-cell analysis, both of which can shed light on tendon cell behavior and fate decisions during the tendon wound healing process.

Tendon is a dense fibrous connective tissue with an abundant extracellular matrix (ECM). Tendon transfers contraction forces (tensile loading) from skeletal muscles to bones, which enables us to move and provides stability to the musculoskeletal system. Tendons have a hierarchical fibrillar arrangement whereby triple-helical type I collagen molecules assemble into fibrils that, in turn, form fibers, fascicles and, ultimately, the tendon unit (1) (Fig. 1). The tendon ECM is mainly composed of type I collagen and a small amount of elastin, surrounded by a hydrated proteoglycan matrix (1). Regularly aligned collagen fibrils serve to withstand tension, and proteoglycans contribute to the viscoelastic properties of the tendon (2). Type I collagen and associated ECM molecules are produced by specialized fibroblastic cells termed tenocytes, which are surrounded by an ECM termed the endotenon (1). Growth factors such as transforming growth factor (TGF)-β and fibroblast growth factors (FGFs) stimulate collagen production in tenocytes (3, 4, 5). Growing evidence indicates that three transcription factors, scleraxis (Scx), mohawk (Mkx), and early growth response 1 (EGR1), play critical roles in regulating collagen synthesis and facilitating tendon development and repair (1). Scx is a basic helix-loop-helix type transcription factor and a specific marker of tenogenic progenitor cells and mature tenocytes (6). Scx plays key roles in tendon development and homeostasis (6, 7, 8). Scx also upregulates tenomodulin (Tnmd), a marker of mature tenocytes (9). There is evidence that, during embryonic development, Scx and sex-determining region Y-box 9 (Sox9) are involved in the generation of tendon progenitors (10) and thus, Scx is the earliest and most persistent marker for the tendon lineage (6, 11). Mkx is a member of the three-amino-acid loop extension superclass of atypical homeobox genes and starts its expression later than Scx in developing tendons (12). Mkx is necessary for the continuation of tendon differentiation once tendon progenitors are initiated during embryonic development, and it is vital for controlling the postnatal growth and maturation of collagen fibrils (13). EGR1 is a zinc-finger transcription factor expressed in many tissues throughout the body, and it is involved in tendon development and tendon repair *via* the induction of Scx and type I collagen (3). Tnmd is a type II transmembrane glycoprotein that is highly expressed in mature tendons and ligaments (14). Tnmd plays a role in the late stages of tendon maturation during development and maintenance by regulating tenocyte proliferation and collagen fibril maturation (14). In this review, we focus on the molecular aspects of tendon development and tendon wound healing.

**Figure 1:**
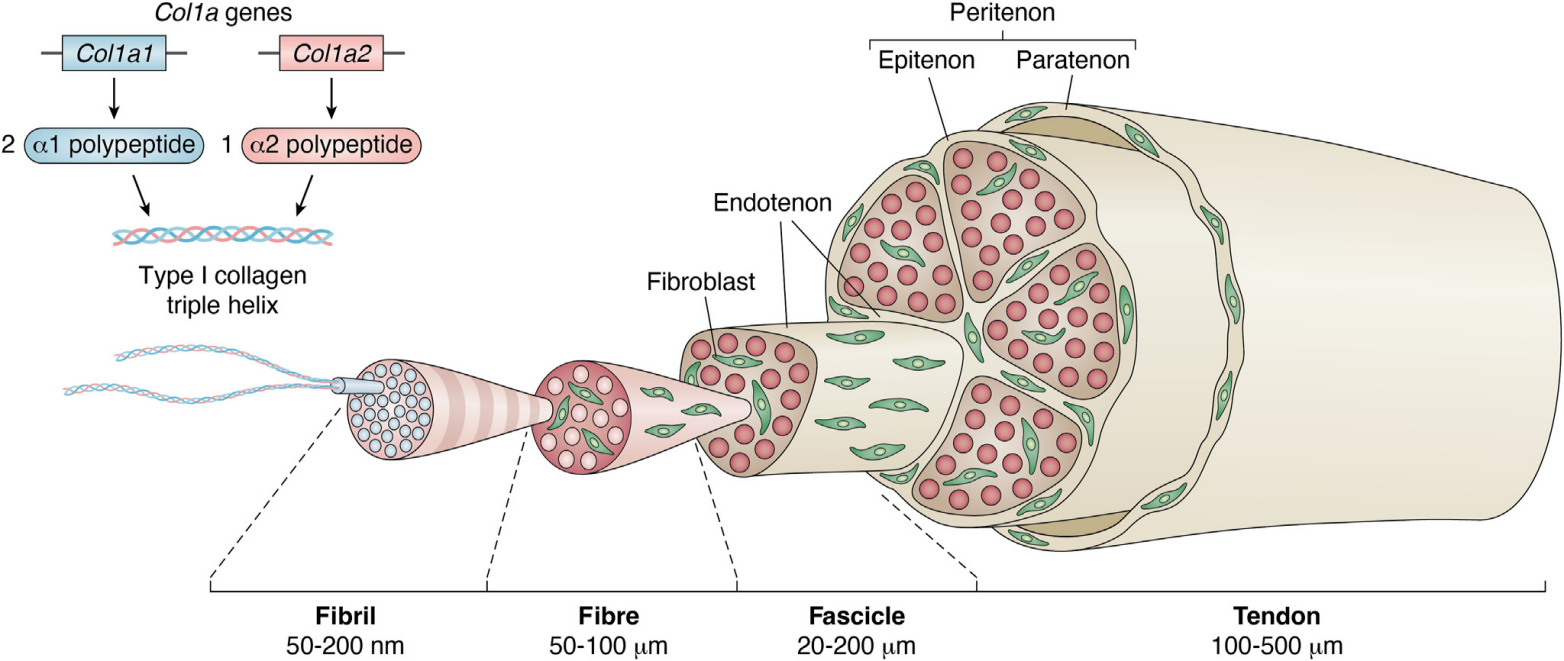
**Hierarchical structure of tendon fascicles (adapted from ref. 1).** Schematic illustration shows the structural composition of tendons from collagen fibrils to the entire tendon. The different layers of the tendon structure, including the endotenon, epitenon, and peritenon (which includes the paratenon), are depicted. Additionally, the involvement of the *Col1a1* and *Col1a2* genes in encoding the α1 and α2 chains of type I collagen, the primary structural component of tendon tissue, is highlighted. Fibroblasts, the primary cellular components responsible for collagen production, are also shown within the fascicles.

Tendon wound healing is a complex biological process that involves both the regeneration of tenocytes and the reconstruction of the ECM, which, like general wound healing, are composed of three sequential and overlapping phases: an inflammatory phase, a proliferation phase, and a remodeling phase (2, 15). The initial inflammatory phase is characterized by extensive cell death at the injury site and the infiltration of inflammatory cells immediately following tendon injury (15). These inflammatory cells produce various cytokines and growth factors, which promote the recruitment of macrophages (16). After a few days, the proliferation phase begins, with cell migration into the injured area, extensive cell proliferation, and the production of collagen fibrils. In this phase, two cellular processes, termed “intrinsic” and “extrinsic”, have been suggested (15): In the intrinsic cellular process, cells derived from both the tendon parenchyma (residential tenocytes) and the peritenon migrate to the wound site and synthesize new ECM which is primarily composed of collagens and glycosaminoglycans (this process plays a central role in tendon wound healing and we therefore describe it in detail later). In the extrinsic cellular process, inflammatory cells such as neutrophils and macrophages are recruited from the circulation (16). The remodeling phase commences approximately 6 weeks after injury, and it is characterized by a decreased cellularity, active type I collagen synthesis and the replacement of type III collagen with type I fibrils (2). Type I collagen is the most abundant collagen and forms thick parallel fibrils in adult tendon tissues, which results in the characteristic high tensile strength and stiffness of tendons (1, 17). Type III collagen forms thinner fibrils than type I collagen and is less rigid (18). Then the initially deposited type III collagen is gradually replaced by type I collagen, which helps restore structural integrity and mechanical strength in adult tendons. It is known that collagen cross-linking is accompanied by tissue fibrosis (19) and that lysyl oxidase family members mainly perform type I collagen cross-linking in tendon wound healing (20). Adult tendon tissue also contains small amounts of other collagen types, including types V and XI, which modify fibril assembly and the mechanical properties of the synthesized collagen fibers (21). After 10 weeks, injured tendons gradually change into fibrous tendon tissue, and this process continues for several years (16).

## Topic 1: Transcription factors and TGF-β signaling in tendon development

### Tendon lineage initiation by Scx

Scx is expressed in the early progenitor cells that are destined to become tendons and ligaments (6). The expression of Scx continues through the various stages of tendon differentiation until the mature tendons are formed (6). This expression pattern of Scx indicates its role in the tendon formation process and its utility as a specific marker for tendons across different developmental stages (6). Recently, useful transgenic reporter mouse lines have been established that express *Scx*-driven GFP as a marker, in which the expression of *Scx*-driven GFP is observed during tendon and ligament development to faithfully recapitulate the endogenous expression of the *Scx* gene in tendon progenitor cells and mature tenocytes (8). These mouse lines could be valuable tools to investigate tendon specification, differentiation, and patterning during development, as well as to analyze the contribution of distinct progenitor-lineage subpopulations to adult tendon wound healing (described in detail later).

Mice lacking the *Scx* gene survive but show a defect in tendon differentiation with a loss of intermuscular tendons and the tendons responsible for transmitting musculoskeletal force in the limbs, tail, and trunk (22). Very importantly, although tendon embryonic progenitors are induced to differentiate even without the *Scx* gene, these progenitors fail to condense into morphologically distinct tendons at embryonic day 13.5 (E13.5) (22). The expression of Tnmd, which acts as a late marker for mature tenocytes, is minimal or absent in tendons and ligaments in *Scx*-null mice (9).

Single-cell RNA sequencing (scRNA-seq) is a powerful tool that allows analysis of the gene expression profiles of individual cells within a heterogeneous population (23). While traditional bulk RNA sequencing (RNA-seq) provides an average gene expression profile of all cells in a sample, it masks the inherent variability among individual cells (23). scRNA-seq enables the identification of distinct cell types, rare cell populations, and transcriptional heterogeneity at the single-cell level (23). scRNA-seq analysis has been performed to analyze tenogenic differentiation induced by TGF-β2 in induced pluripotent stem cells (iPSCs) established from ScxGFP transgenic mice. This analysis revealed 11 distinct clusters, and a progressive trajectory where cells shift from Scx+/Tnmd− to Scx+/Tnmd+ and finally to the Scx−/Tnmd+ state (24), indicating that a similar dynamic trajectory occurs *in vivo* during tendon development. In a comprehensive study on the molecular mechanisms of Scx in tendon development and homeostasis, a novel Scx^*Flag*^ knock-in mouse model was used to precisely map Scx binding sites through chromatin immunoprecipitation, followed by high-throughput DNA sequencing (25). A total of 7520 genes is associated with Scx-binding genomic regions, and comparative analysis with previously reported embryonic tendon cell RNA-seq data further identified 490 Scx direct target genes critical for tendon development. In the Scx knockout (KO) mouse model, RNA-seq was used to identify 68 genes that are dependent on Scx function. Furthermore, using *in situ* hybridization and quantitative real-time PCR, this study validated the expression of Scx-dependent genes such as *Fmod*, *Kera*, *Htra3*, *Ssc5d*, *Tnmd*, and *Zfp185*, all of which are crucial for tendon cell differentiation and/or collagen fibrillogenesis (25). Ectopic overexpression of *FLAG-Scx* in human mesenchymal stem cells (MSCs) leads to a more spread cellular morphology, reduces the proliferation rate and decreases clonogenicity compared to the control, suggesting a role of Scx in directing the differentiation of MSCs into tendon progenitors (26). This experimental evidence documents that Scx plays a pivotal role in guiding the differentiation of progenitor cells into tenocytes.

### Role of Mkx in tendon development

Next, we discuss Mkx, focusing on the functional differences between it and Scx. Robust Mkx mRNA expression has been identified in the differentiating mouse tendons of the limbs and tail at E13.5 and E14.5, stages at which the tendon progenitors undergo condensation and differentiation (13). Mkx^−/−^ mice exhibit a reduction in tendon mass and size, collagen fibril diameter, and type I collagen production, but there is no significant difference in cell numbers within the tail tendon fiber bundles compared to WT mice (27). Tenogenic gene expression patterns and collagen fibril diameters were compared between Mkx-overexpressing mouse-derived MSCs and Scx-overexpressing ones *in vitro* (28). Mkx-overexpressing MSCs show significantly increased expression of critical tenogenic genes such as *Col1a1*, *Fmod*, and *Tnmd*, and a significantly larger diameter of the collagen fibrils than Scx-overexpressing MSCs, suggesting that Mkx is more potent in driving tenogenesis compared to Scx (28). Thus, Mkx plays a crucial role in tendon development by promoting tenocyte differentiation and collagen fibrillogenesis.

### Functional difference between Mkx and Scx during embryogenesis

There is obviously a functional difference between Mkx and Scx in tendon development. Mkx^−/−^ mutant mice do not display any obvious defects in the cartilage, bone, skeletal muscle, or tendon, indicating that the absence of Mkx does not critically impair the overall structure or integrity of the skeletal system (13). In contrast, Scx^−/−^ mutant mice exhibit severe tendon defects, including the loss of segments of tendons or complete tendons, particularly affecting the force-transmitting tendons (22). This distinction indicates that Scx plays essential roles in the initial commitment of mesenchymal cells to the tendon lineage and the early formation of tendon structures, whereas Mkx reinforces and maintains the ECM once tendon identity has been established. Thus, it is argued that the expression of Scx and that of Mkx in tendon cells are not interdependent during embryogenesis. However, these transcription factors interact during tendon maturation. Scx expression is maintained in Mkx^−/−^ mouse embryos, and, conversely, Mkx expression is maintained in Scx^−/−^ mouse embryos (13), indicating that the expression of *Scx* and that of *Mkx* are upregulated by different pathways. Distinct roles of Mkx and Scx are particularly noted in the upregulation of type I collagen production (27, 29). Scx upregulates the expression of the type I collagen gene in mice by binding to specific *cis*-acting elements in the *Col1a1* promoter region, particularly tendon-specific element 2 (TSE2) (29). In spite of the decreased expression of type I collagen in Achilles tendons in *Mkx*-null mice, these tendons in *Mkx*-null mice show increased expression of *Scx* (27). These results suggest that there exists a sensor that recognizes the expression level of the type I collagen gene. The expression of Scx and Mkx is regulated by different mechanisms, in which Scx could be upregulated in a compensatory manner when Mkx is missing. Alternatively, there could be a negative modulator in the type I collagen gene, which is suppressed by Mkx under normal conditions. It remains to be elucidated whether Mkx directly upregulates the type I collagen gene or suppresses repressors of this gene.

### Role of TGF-**β** in tendon development

Recently published observations provide evidence that TGF-β signaling is crucial for tendon development (5, 30, 31, 32). TGF-β signaling is a potent inducer of the tendon progenitor marker *Scx* in mouse tendon progenitor cells in both organ culture and cell culture, and the disruption of TGF-β signaling utilizing *Tgfb2* and *Tgfb3* double-null or type II TGF-β receptor (*Tgfbr2*)-null mice results in the loss of most tendons and ligaments (5). The loss of tendons and ligaments is first apparent at the stage when tendon progenitors are recruited and positioned between the differentiating muscles and cartilage, suggesting that TGF-β signaling is essential for the recruitment and spatial organization of these cells (5). During the development of mouse limbs, the expression levels of *Scx* and *Col1a1* in MSCs are upregulated approximately 8- and 3-fold, respectively, through the activation of TGF-β/SMAD2/3 signaling, but are inhibited by the blockade of TGF-β receptors using the small molecule SB43 or the blockade of SMAD2/3 activity using SIS3, an inhibitor of Smad3 phosphorylation, precisely dissecting the functional significance of TGF-β/SMAD2/3 signaling in driving undifferentiated mesodermal cells toward the tendon lineage (31). An elegant study has demonstrated that, when the *Tgfbr2* gene is genetically disrupted in mice, tendon development is not disrupted (32). However, shortly after the birth of the mice, tenocytes show a gain of progenitor-like phenotypes such as the expression of stem cell antigen-1 (*Sca-1*) and cluster of differentiation 44 (*CD44*), and also show a loss of tendon markers such as *Scx*, *Fmod*, *Tnmd*, and *Col1a1* (32). Interestingly, those progenitor-like phenotypes are reversed by the reintroduction of the *Tgfbr2* gene using a viral vector (32). Thus, this study nicely highlights a continuous and essential role of TGF-β signaling in the maintenance of tendon cell fate.

## Topic 2: Fibrotic tendon healing

Fibrosis is not a disease but an outcome of a tissue repair response to tissue injury and is defined as an excessive accumulation of ECM components (33). Extensive and persistent fibrosis triggers chronic fibrotic disease, which is associated with increased numbers of myofibroblasts (34). Fibrosis is reversible under some circumstances, and the mechanism of fibrosis resolution encompasses the elimination of fibrogenic myofibroblasts (35). Tendon injury often results in the formation of fibrotic scars with the deposition of excessive, disorganized ECM rather than regeneration of the native tendon structure following injury (36). This is problematic because the healed tendon remains weaker and less elastic in the long term (37), which can lead to a permanent reduction in performance (38) and impaired quality of life (15). Currently, the main barrier to designing novel treatment strategies is our insufficient understanding of the mechanisms responsible for cellular contributions to adult tendon injury repair and the reorganization of the tendon ECM network following injury.

### Roles of Scx, Mkx, and TGF-**β** signaling in adult tendon wound healing

The roles of Scx in tendon development and adult tendon wound healing are closely related, since the cellular process regulated by Scx during embryogenesis is reactivated during adult tendon repair. For instance, in a murine model of patellar tendon injury, the expression of Scx significantly increases during the repair process, particularly in the proliferative and remodeling phases, compared to the uninjured tendon (39). This reactivation of Scx coordinates the upregulation of tendon-related genes such as *Col1a1* and *Tnmd*, indicating that Scx plays a role in orchestrating the healing response. In adult mouse tendon wound healing, Scx-expressing cells are recruited to the injury site, where they play a critical role in repairing the damaged tissue (40, 41, 42). These cells contribute to tissue repair by synthesizing collagen and reorganizing the ECM, both of which are essential for restoring the mechanical properties of the tendon (42). While the specific behaviors and migration patterns of Scx-expressing cells during adult tendon healing are complex, as discussed in detail in later sections, the ability of Scx-expressing cells to promote collagen production and ECM remodeling during adult tendon healing mirrors their roles during tendon development.

Mkx is involved in the promotion of adult tendon wound healing. Mkx-expressing MSC sheets transplanted into a surgically created mouse Achilles tendon defect display histological maturation: the Achilles tendon defect regenerates like typical mature tendon tissues characterized by the formation of collagen fibrils with an increased diameter and small crimped patterns, as well as tenocytes with a pronounced spindle-shaped morphology (28). Furthermore, tendons with Mkx-expressing MSC sheets show better biomechanical properties than those with control cell sheets. This observation highlights the significant regenerative potential of Mkx in tendon wound healing by promoting tenogenic differentiation in MSCs (28). Thus, the role of Mkx in adult tendon healing through collagen fibril maturation mimics its function during tendon development.

TGF-β signaling promotes the proliferation, migration, and differentiation of tendon stem/progenitor cells (TSPCs) during the adult tendon healing process, all of which recapitulates its functions during tendon development. TGF-β/SMAD signaling induces the differentiation of TSPCs into Scx-expressing tenocytes and their migration from the paratenon to the wound site following mouse tendon injury *in vivo* (42). Furthermore, TGF-β/SMAD2/3 signaling accelerates the proliferation and migration of rat-derived TSPCs *in vitro* (43). In neonatal mouse tendon, TGF-β signaling is essential for recruiting tenogenic cells derived from both Scx-lineage and non-Scx-lineage cells to the injury site (44). In adult tendon tissue, a specific region where non-Scx-lineage cells exist and are recruited to the injury site is known as the peritenon (40, 42, 45). *ScxCre* driver-mediated lineage tracing in *Tgfbr2* mutant mouse tendon reveals that mutant tendons show progressive matrix deterioration or degeneration, a characteristic feature of tendinopathy and spontaneous tendon rupture (46). Mechanistically, the recruited cells are not derived from the peritenon or tendon sheath, but instead they show a Sox9-expressing lineage originating outside the peritenon or tendon sheath (46). Therefore, it would be quite valuable to identify the phenotypic changes in response to tendon wound healing in *Tgfbr2* mutant mice. This knowledge could provide insights into potential therapeutic targets for enhancing tendon repair.

### Lineage cell tracing of TSPCs in tendon healing

#### Isolation of TSPCs from tendon parenchyma

Pioneer work by Bi *et al.* (47) has identified TSPCs as a small population of cells from the tendon parenchyma in humans and mice. The properties of TSPCs include clonogenicity, self-renewal capacity, and multipotency (adipogenic, osteogenic, and chondrogenic). Interestingly, depletion of biglycan and fibromodulin, which are known to organize the unique composition of the ECM niche, directly inhibits the tenogenic differentiation of mouse-derived TSPCs through signaling *via* bone morphogenetic protein (BMP) in both *in vitro* and *in vivo* models (47). TSPCs express a variety of phenotypic markers that are characteristic of MSCs, including Sca-1, cluster of differentiation (CD) 44, CD90, CD90.1, CD90.2, CD105, stromal precursor antigen-1 (Stro-1), CD146, nucleostemin, octamer-binding transcription factor 4 (Oct-4), and stage-specific embryonic antigen-4 (SSEA-4), but are negative for CD31, CD34, CD18, CD117, CD45, fetal liver kinase-1 (Flk-1), CD144, and CD106 (reviewed in (48)). One issue is that there are no specific markers to distinguish TSPCs from tenocytes, leading to inconsistencies in research findings and impeding the accurate characterization of these cell types (49). In the future, advanced technologies such as single-cell genome and transcriptome analysis could identify gene clusters that can distinguish TSPCs from tenocytes and other nonhematopoietic adult stem cells.

#### Effect of TSPCs on tendon wound healing

An *in vitro* study comparing rat-derived TSPCs with bone marrow stromal cells (BMSCs) shows that TSPCs have a higher clonogenicity, greater proliferative capacity, and superior potential for differentiation into osteogenic, chondrogenic, and adipogenic lineages compared to BMSCs, suggesting that TSPCs could be a more promising cellular source than BMSCs for musculoskeletal tissue regeneration (50). When the regenerative ability of TSPCs was assessed by transplantation into rat tendon wounds *in vivo*, the transplanted TSPCs significantly enhanced tendon healing by promoting collagen production, and improving cellular and collagen fiber alignment as well as biomechanical properties such as tensile strength and elasticity in a rat patellar tendon window defect model (in which the central portion [∼1 mm] of the patellar tendon is removed) (51). Allogeneic rat-derived TSPCs transplanted into the rat patellar tendon wound promote tendon healing and exhibit weak immunoreactions and antiinflammatory effects, but the transplanted TSPCs found within the wound up to 2 weeks disappear by 4 weeks post injury (52). In another study, GFP-labeled TSPCs transplanted into rat Achilles tendon defects were well aligned in the injured ECM, and actively expressed Tnmd at 4 weeks after the injury, suggesting that the transplanted TSPCs were integrated into the tendon defect and had differentiated into mature tenocytes (53). In addition, a study was conducted using mouse iPSCs that received both basic fibroblast growth factor (bFGF) and TGF-β1 to induce differentiation into tenocyte-like cells (54). When these iPSC-derived tenocyte-like cells were transplanted into injured mouse tendons, the tenocyte-like cells expressed *Fgf2*, and regenerating tendons showed higher expression of bFGF than control tendons, as well as reduced scar formation, suggesting that bFGF can promote tendon healing *via* a paracrine mechanism (54). These findings indicate that transplanted TSPCs differentiate into mature tenocytes by themselves, as well as promoting tendon repair through indirect mechanisms, *i.e.*, the production of paracrine factors and suppression of the inflammatory response.

#### Distribution and characteristics of stem/progenitor cells in adult tendon tissue

The unique feature of adult tendon tissue is that the stem and progenitor cell populations are distributed not only within the main body of the tendon but also in the surrounding peritendon area (45, 55, 56). Stem and progenitor cells derived from both the tendon parenchyma and the peritenon of mice (45) and horses (55) have the potential to differentiate into adipogenic, chondrogenic, osteogenic, and tenogenic lineages. These cells can form tendon-like tissues rich in collagen fibrils *in vitro* that are similar to embryonic tendon (56). Residential tenocytes from mouse tendon show higher levels of tendon markers, while peritendon-derived cells from the same mouse tendon express more vascular markers, yet both are multipotent and capable of forming tendons (45). Peritendon-derived cells from horse tendon show faster migration, a higher replication rate, and a greater propensity to differentiate toward a myofibroblastic phenotype than cells derived from tendon proper from the same horse (55), suggesting that they could provide a rapid response to injury and offer a more nutrient-rich environment (57, 58). Therefore, peritendon-derived stem and progenitor cells could migrate to the injury site and contribute to tendon repair in collaboration with residential tenocytes.

#### Contribution of Scx-positive TSPCs to tendon healing

Scx-positive TSPCs originate primarily from progenitor cells located within the tendon tissue and surrounding areas such as the paratenon. To investigate the contribution of these cells to tendon wound healing, lineage-tracing analysis utilizing *Scx*-GFP transgenic mice is a valuable tool for unraveling the complex processes involved in tendon wound healing (8). This transgenic strain allows us to directly monitor functions in target cells, *i.e.*, how they differentiate, mobilize, migrate, and play their roles at tendon wound sites (Table 1). We have recently documented the contribution of progenitor cells to adult tendon wound healing using a novel tendon partial transection model in *Scx*-GFP transgenic mice (42). In response to tendon injury, a subset of Sca-1 positive and Scx-negative progenitors in the paratenon region display induction of Scx, which is initiated by TGF-β signaling. These cells migrate to the wound site and play a central role in the repair of injured tendon. A surprising finding is that Scx-expressing residential tenocytes exhibit a delayed response, *i.e.*, migrating to the wound site at a later stage (42).

**Table 1:** Lineage tracing studies in adult mouse tendon wound healing

Injury type	Outcomes	Tracing marker(s)	Effector	Ref.
Central third patellar tendon injury	α-SMA+ paratenon cells migrate to the wound site and differentiate into Scx+ cells	α-SMA, Scx	Collagen, tenascin-C	(57)
Achilles tendon partial resection	Scx+ cells migrate to the wound site and produce ECMs to bridge the defect	Scx	Type I collagen, type III collagen	(42)
FDL tendon complete transection with surgical repair	Scx+ cells are found in the organized bridging tissue and S100a4+ cells are localized throughout the entire scar region	Scx, S100a4	Myofibroblast	(59)
Central third patellar tendon injury	Nestin+/Scx+ cells in endotenon/peritenon migrate to the wound site	Nestin	Collagen, *Mkx*, *Scx*	(64)
Patellar tendon punch injury	Tppp3+Pdgfra+ cells migrate to the wound site and differentiate into Scx+ cells	Tppp3, Pdgfra	Fibromodulin, tenascin-C	(63)
FDL tendon complete transection with surgical repair	S100a4-lineage cells represent α-SMA+ myofibroblasts in scar tissue	S100a4	Myofibroblast	(62)
Achilles tendon complete transection	Scx+ tenocytes do not contribute to healed scar tissue	Scx	Type I collagen, type III collagen	(41)
FDL tendon complete transection with surgical repair	Scx-lineage cells are not required for the formation of a bridging collagen matrix	Scx	Myofibroblast, collagen	(60)

α-SMA, alpha-smooth muscle actin; FDL, flexor digitorum longus; Pdgfra, platelet-derived growth factor receptor α; Tppp3, tubulin polymerization-promoting protein family member 3.

Another study reported a unique Scx-lineage tracing investigation using a murine complete tendon transection repair model. To label and compare the Scx-lineage subpopulation, mice received a Cre-inducing tamoxifen injection on days 0 to 2 following injury (immediately after injury; termed Scx^0-2^) or days 5 to 7 following injury (early proliferating stage; termed Scx^5-7^) (59). Interestingly, at 14 days post injury, whereas no Scx^0-2^ cells were found within the scar tissue, Scx^5-7^ cells were localized within the scar tissue at the injury site. Thus, there exist different induction schemes in Scx-lineage subpopulations. Taken together, these results suggest that paratenon-derived Scx-positive TSPCs initiate the repair process and contribute to the early stages of tendon healing, whereas tendon parenchyma derived Scx-positive residential tenocytes participate in the later stages of tendon healing and play a role in ECM remodeling.

Very recently, a study was carried out to define the function of Scx-lineage cells during adult tendon repair (60). Using Scx-Cre and Rosa-(diphtheria toxin receptor) DTR^Loxp-stop-Loxp(LSL)^ (ScxLin^DTR^) mouse models, Scx-lineage cells were depleted by local injections of diphtheria toxin prior to complete tendon transection (approximately 57% depletion of Scx-lineage cells in uninjured tendon) (60). Surprisingly, depletion of Scx-lineage cells resulted in improved biomechanical properties without impairment of the tendon’s range of motion (gliding function) at 28 days post injury and did not disrupt the formation of a bridging collagen matrix. Importantly, in the healing tendon, a significant decrease in the proportion of Scx-lineage cells was found in ScxLin^DTR^ compared to the WT at 28 days post surgery, whereas no significant difference was observed at 14 days post injury. In addition, ScxLin^DTR^ healing tendon showed significantly increased alpha-smooth muscle actin (αSMA)+ myofibroblasts compared to the WT at 28 days post injury, suggesting that αSMA+ myofibroblasts can contribute to the improved biomechanical properties observed in the ScxLin^DTR^ healing tendon. However, there are several issues with this model system. First, nearly 40% of Scx-lineage cells remain intact prior to tendon injury. Second, the present ScxLin^DTR^ ablation system targets cells prior to injury and thus does not count any cells that turn on Scx after injury. An additional report by the same group reveals that the depletion of Scx-lineage cells between 14 and 18 days post injury in the same mouse model results in substantial impairment of mechanical properties and collagen fibril maturation in healing tendon at 28 days post injury (61). However, a striking finding is that this stagnation is transient: the structural and biomechanical properties of ScxLin^DTR^ tendon are not significantly different from WT repairs at 56 days post injury (61).

We have also investigated the role of Scx in tendon healing using a partial tendon transection model in *Scx*-null mice (42). In WT mice, Sca-1-positive progenitor cells migrate to the wound site. In the *Scx*-null wound, however, although Sca-1-positive progenitors migrate to the lesion site, they impair the ability of the ECM assembly to bridge the defect. As a result, *Scx*-null wounds form cartilage-like tissues that develop ectopic ossification. Mechanistically, *Scx*-null progenitors exhibit a higher chondrogenic potential with upregulation of the Sox9 coactivator PPAR-γ coactivator-1α (PGC-1α), and knock-in of full-length *Scx* significantly inhibits *Sox9* (42). These observations clearly demonstrate that the cells within the paratenon, which do not express Scx, do respond to injury by upregulating tenogenic markers, and proliferate and migrate to bridge the defect (40). These dynamic responses highlight the plasticity of these cells in adult tendon repair.

#### What can we learn from different experimental systems when lineage-tracing analyses focusing on Scx-expressing cells are carried out in tendon wound healing?

As highlighted above, several lineage-tracing studies using mouse models have specifically focused on the Scx-expressing cell population to investigate its behavior and contribution to adult tendon wound healing (41, 42, 57, 59). In response to partial tendon transection injury but not to complete transection injury, cells located in the paratenon or epitenon migrate to the wound site and turn on Scx during the tendon healing process (42, 57). In the case of complete tendon transection, however, this does not happen: the complete-transection model shows fibrotic scar healing with no migration of Scx-expressing cells in the gap between the tendon stubs at 14 days post injury (41). Furthermore, cells expressing extrinsic αSMA persist at the wound site and form a permanent scar, which in turn does not induce the migration of Scx-expressing cells to the wound site.

**Figure 2:**
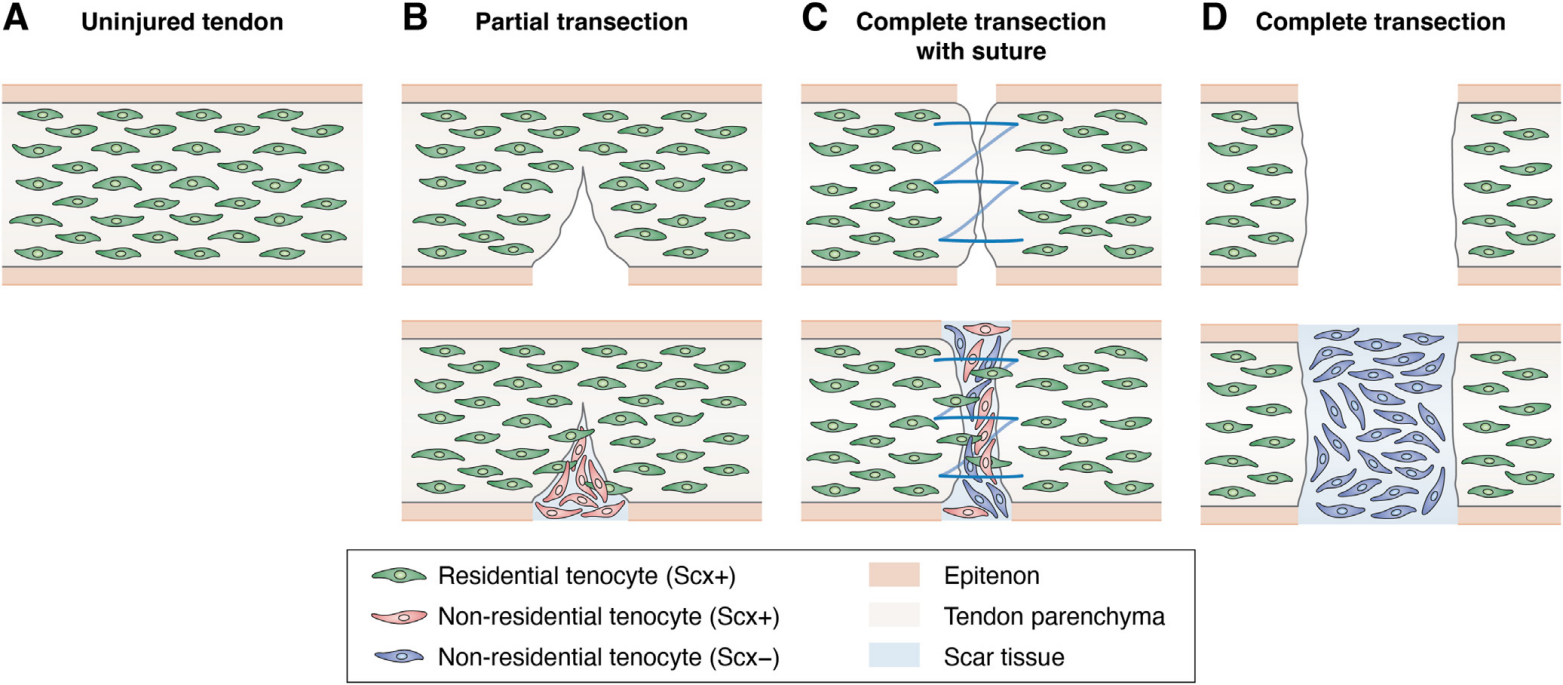
**Schematic illustration of three different wound healing models at 14 days following injury in adult mouse Achilles tendon.***A*, uninjured tendon. *B*, partial transection. Just after injury, the tendon edge is still intact and has connections (*upper* panel). At 14 days following injury, nonresidential tenocytes expressing Scx are present at the wound site *(lower* panel). At a later stage, residential tenocytes expressing Scx migrate to the wound site as a minor population. *C*, complete transection with suture treatment. Just after transection, sutures create a certain tension between the ends of the transected tendon (*upper* panel). At 14 days following injury, nonresidential tenocytes both with and without Scx expression and residential tenocytes expressing Scx are present at the wound site (*lower* panel). *D*, complete transection. Just after injury, the tendon ends are completely separated from each other (*upper* panel). At 14 days following injury, only nonresidential tenocytes not expressing Scx are present at the wound site (*lower* panel). Scx, scleraxis.

We can now argue for the following scenario: In complete tendon transection models, transected tendon ends are separated from each other and therefore mechanical force within the tendon is completely lost. This leads to a delay in the migration of tenocytes and TSPCs, which often results in more disorganized ECM deposition and fibrotic scar tissue with poor mechanical properties. In contrast, in partial tendon transection models, because the tendon is not completely transected, mechanical force is retained to some extent. Under these conditions, tenocytes and TSPCs can migrate more efficiently, resulting in repair with better aligned collagen fibers and improved mechanical properties. In specific injury models such as complete tendon transection with surgical treatment (sutures), the sutures create a certain tension between the transected tendons, and mechanical force is reestablished to some extent (Fig. 2). However, in contrast to partial tendon transection models, the healing outcome often results in the formation of fibrotic tissue.

The lineage cell tracing systems described above provide insights into how progenitor cells process dynamic functions during tendon wound healing. The *Scx*-GFP transgenic reporter mouse system (8) has been extensively utilized in various studies, including some involving lineage cell tracing. However, considering the whole process of adult tendon wound healing, it is important to note that to track Scx alone as a marker would not be sufficient to distinguish so many subpopulations in the healing process. Some other subpopulations have recently been identified and their role in tendon wound healing clarified (62, 63, 64). The novel tracing markers S100a4, nestin, and Tppp3/Pdgfra, enable us to determine the extent to which each subpopulation can differentiate into mature tenocytes and contribute to ECM reorganization following injury.

##### S100a4

S100a4 has recently been identified as a key regulator of fibrotic tendon healing (62). S100a4, a member of the S100 family of EF-hand Ca^2+^-binding proteins, can drive fibrosis in various tissues (65, 66, 67), and it is known to be expressed in residential tenocytes (62). S100a4+ cells differentiate into α-SMA+ myofibroblasts, which are responsible for fibrotic tissue formation during the healing process (62). Lineage-tracing analysis in the mouse healing tendon (59, 62) shows that a large portion (∼70%) of α-SMA+ myofibroblasts is derived from S100a4-lineage cells. Importantly, these S100a4-lineage cells gradually lose the expression of S100a4 when they transdifferentiate into α-SMA+ myofibroblasts. These transdifferentiation events highlight the unique and dynamic nature of cellular phenotypes during tendon wound healing.

To investigate the relationship between Scx+ and S100a4+ cells in an adult murine flexor tendon complete transection repair model, dual-tracing analysis of Scx and S100a4 was carried out (59). At day 14 to day 21 post injury, Scx-lineage cells were mainly localized in the bridging tissue (the center of the injured region), whereas S100a4-lineage cells were found in the entire scar tissue region (not only toward the center but also at the periphery of the injured region). Interestingly, the Scx-lineage cell population in the bridging tissue was greater than the S100a4+ cell population (*p* < 0.01) at day 21 post injury. Thus, these data define a heterogenous environment of the adult tendon and suggest different roles for each subpopulation during adult tendon wound healing.

Intrinsic Scx-lineage cells contribute to organized bridging ECM following tendon injury, whereas S100a4+ cells contribute to scar formation. Although the depletion of S100a4+ cells has been shown to impair the restoration of biomechanical properties during murine flexor tendon healing, S100a4 haploinsufficiency (a 50% reduction in S100a4 expression) promotes the deposition of a mature collagen matrix and significantly (∼35%, *p* = 0.003) increases the maximum load at failure compared to the WT in healing tendons (62). Indeed, S100a4 is known to stimulate the inflammatory response with the recruitment of macrophages and to promote the fibrotic healing process. S100a4 haploinsufficiency results in decreased numbers of iNOS-expressing macrophages (proinflammatory) in the healing tendon compared to the WT, whereas this haploinsufficiency makes no difference in IL1ra-expressing macrophages (antiinflammatory) compared to the WT. These findings suggest that an appropriate expression level of S100a4 is necessary to achieve an optimal tendon healing outcome and to avoid excessive fibrosis.

##### Nestin

A subpopulation of TSPCs expressing nestin has been identified and characterized through the single-cell analysis of adult human tendon tissue (64). Nestin is a type VI intermediate filament protein that was initially identified in neural stem cells and was subsequently found in various types of stem and progenitor cells in different tissues. Nestin-expressing TSPCs in the mouse show a robust self-renewal capacity and upregulated expression levels of *Scx*, *Mkx*, *Eln*, *Col I*, and *Col XIV* (64). Nestin-expressing TSPCs that coexpress Scx are predominantly localized in the endotenon and peritenon in normal adult tendon in both humans and mice. However, following tendon injury in the mouse, nestin-expressing TSPCs accumulate at the wound site, and their numbers peak 1 week after injury, gradually decreasing thereafter. Knockdown of *nestin* in a rat patellar tendon defect model results in less aligned collagen arrangements (*i.e.*, reduced histological maturity) and impaired biomechanical properties in the healing tendon (64). It is interesting to speculate that these cells serve as a potential reservoir of stem/progenitor cells. The unique characteristics and regenerative potential of nestin could provide a promising approach to future therapeutic interventions in tendon-related pathologies.

##### Tppp3/Pdgfra

Cells expressing tubulin polymerization-promoting protein family member 3 (Tppp3) have been identified as potential tendon stem cells in a mouse model using scRNA-seq (63). *Tppp3*-expressing cells are predominantly present in the mouse tendon sheath and remain quiescent. In response to tendon injury, they are activated through platelet-derived growth factor (PDGF) signaling and migrate to the tendon wound site. Since there is evidence that 50.6% of *Tppp3*-expressing cells coexpress platelet-derived growth factor receptor α (*Pdgfra*), and that cells expressing both *Tppp3* and *Pdgfra* (*Tppp3*^*+*^*Pdgfra*^*+*^) contribute to tendon regeneration in a mouse patellar tendon punch-injury model (63), the interaction between *Tppp3*^*+*^*Pdgfra*^*+*^ cells and PDGF signaling was investigated. It was shown that PDGF-AA (concentration range: 0.5–5.0 ng/ml), a ligand for PDGFRα, promotes the differentiation of these double-positive cells into tenocytes. Unexpectedly, a *Tppp3*^*-*^*Pdgfra*^*+*^ fibro-adipogenic progenitor subpopulation was found to coexist in the tendon stem cell niche and give rise to fibrotic cells, indicating a clandestine origin of fibrotic scars in healing tendons. This observation could explain why fibrotic scar formation often occurs in injured tendons. These findings provide important evidence that PDGF signaling can affect not only the extent of differentiation of *Tppp3*-expressing stem cells into tenocytes but also the involvement of *Tppp3*-expressing fibrotic cells in fibrotic scar formation following tendon injury.

### Heterogeneity of Scx-lineage cells in fibrotic tendon healing

#### Heterogeneity of cellular composition in tendon fibroblasts

An informative study using scRNA-seq analysis by De Micheli *et al.* (68) indicates the existence of heterogeneity in the cellular composition of mouse uninjured native tendon, and 11 distinct cell types, including three previously uncharacterized subpopulations of tendon fibroblasts, were identified. These three tendon fibroblast subpopulations are termed (1) tendon fibroblasts 1; (2) tendon fibroblasts 2; and (3) junctional fibroblasts. All three subpopulations express type I collagen at a moderate to high level but have distinct gene expression profiles. Tendon fibroblasts 1 predominantly express osteopontin (*Spp1*), which plays roles in cell adhesion and migration, and thus potentially respond to tendon injury and repair. Tendon fibroblasts 2 highly express dermatopontin (*Dpt*), which plays roles in collagen fibril organization, and thus could potentially stabilize the structure of the tendon matrix. Junctional fibroblasts highly express type XXII collagen (*Col22a1*), and these cells are specifically localized to the adult myotendinous junction, and thus potentially maintain the structural integrity and function of the tendon at junctional sites (69, 70). The same article (68) also shows that only a minority of adult mouse tendon fibroblasts expresses Scx, although our previous findings in the *Scx*-GFP transgenic reporter mouse model and in human ligament immunostaining using our anti-Scx antibody clearly indicate widespread expression of Scx in tendon/ligament fibroblasts (71, 72). This discrepancy may result from methodological differences. In the study by De Micheli *et al.* (68), the expression of Scx was evaluated using scRNA-seq and RNA *in situ* hybridization. Both techniques could miss transiently or weakly expressed genes, which may lead to an underestimated expression level. Regardless, when one analyzes how a certain molecule contributes to tendon wound healing, it is important to take account of the experimental model systems and methodologies used.

A multiomic single-cell study (73) identified and characterized five distinct populations of *COL1A1/2*-expressing tenocytes in healthy and injured human tendons. These distinct populations include (1) *KRT7/SCX*+ cells, coexpressing genes associated with extracellular tendon microfibrils (*FBN1*, *MFAP5*, *VCAN*, and *EMILIN1*); (2) *PTX3*+ cells, coexpressing high levels of proinflammatory markers; (3) *APOD*+ fibro-adipogenic progenitors; (4) *TPPP3/PRG4*+ chondrogenic cells; and (5) *ITGA7*+ smooth muscle-mesenchymal cells. These five subpopulations are found in both healthy and injured tendon, but injured tendon shows increased proportions of *PTX3*+ cells, *APOD*+ fibro-adipogenic progenitors and *TPPP3/PRG4*+ chondrogenic cells compared to healthy tendon. Another study (74) reveals two distinct populations of tendon fibroblasts based on their spatial localization and gene expression profiles in the healthy (uninjured) rat patellar tendon. The first population of these tendon fibroblasts is located in the central portion of the tendon tissue and is involved in the deposition of type I collagen in response to mechanical loading. The second population is located in the peripheral portion, and coexists with red blood cells, pericytes, and immune cells.

Thus, the discovery of novel tendon subpopulations that have different phenotypes between the normal and injured states could have a benefit for new therapeutic approaches in tendinopathy.

#### Spatial and temporal contribution of different Scx-lineage cells to fibrotic tendon healing

Scx-lineage cells show heterogeneity during the healing process in response to injury. Ackerman *et al.* (75) demonstrated temporal changes in Scx-lineage cells during mouse tendon healing utilizing a combination of lineage cell tracing and spatial transcriptomic profiling: Scx-lineage cells are classified into six clusters: namely, C0^synthetic^, located in less reactive areas further from the injured site; C1^native^^_tendon^, located in uninjured tendon; C2^reactive^, located adjacent to the injured site; C3^fibrotic^, located at the periphery of the injured site; C4^inflammatory^, located at the center of the injured site and adjacent to C2; and C5^muscle-assoc^, representing the muscle–tendon interface. These different clusters of Scx-lineage cells occupy distinct locations or niches within the tendon, which can influence their response to injury and fibrosis. In these clusters, C1 and C5 represent the uninjured tendon, and C0, C2, and C3 represent the healing tendon. Pseudotime analysis reveals that C1 can differentiate toward C0, C3, or C2: the first synthetic trajectory (C0), characterized by genes such as *Tnmd*, *Col1a1*, and *Fmod*, supports tenogenesis and ECM organization in tendon regeneration. The second fibrotic trajectory (C3), enriched in profibrotic markers such as *Col3a1*, *Postn*, and *Thbs3*, indicates a high level of ECM synthesis toward scar formation. The last reactive trajectory (C2), characterized by genes such as *Mmp13*, *Lox*, and *Fbln2*, relates to ECM organization, collagen catabolic processes, and the positive regulation of many cellular activities such as cell adhesion, migration, proliferation, and collagen biosynthesis. The transition toward one of these fates (trajectories) is necessary for dynamic tendon repair. The transition from C1 to C2 is critical for effective tendon healing, since this transition initiates the healing process by promoting inflammation and ECM organization. Key transcription factors such as *Srf*, *Sp1*, *Egr1*, *Fosl1*, *Smad3*, and *Klf4* regulate that differentiation trajectory. However, the persistent presence of C2 cluster cells prevents the completion of the healing process. Thus, fate-switching C2 to C0 is the key to restoring tendon structure and function. These findings suggest that the subpopulation of Scx-lineage cells has a temporal and spatial distribution pattern, and that understanding these temporally and spatially dependent molecular programs and cellular interactions could help to identify novel targets for treatment.

The recent observations described above have now provided compelling evidence that the Scx-expressing progenitor cell subpopulation is heterogeneous in tendon healing, and that this heterogeneity affects the progression and outcome of fibrotic tendon healing (59, 60, 75).

The heterogeneity of Scx-lineage cells in fibrotic tendon healing represents the following characteristics.

(1) Functional diversity: A certain subset of adult Scx-lineage cells has a higher propensity to differentiate into αSMA+ myofibroblasts. These are contractile cells responsible for scar formation in fibrosis. Other subsets have a lower potential for differentiation into myofibroblasts and instead contribute to different processes such as immune regulation or ECM remodeling (59, 75).
(2) Spatial distribution: Pseudotime trajectory analysis suggests that Scx-expressing cells will not be uniformly distributed in the tendon but will form local clusters (subsets) with distinct functional roles (75).
(3) Dynamic changes in cellular phenotypes over time: The heterogeneity of Scx-lineage cells in fibrotic tendon healing is not static but can evolve during different phases of the healing process. In the initial stage, Scx-lineage cells predominantly exhibit inflammatory characteristics. As healing progresses, Scx-lineage cells shift toward a more synthetic phenotype and mainly perform ECM synthesis. At a later stage, some Scx-lineage cells adopt a fibrotic phenotype to contribute to scar tissue formation, and others continue to participate in ECM remodeling. Detailed time-course analyses in future studies of molecular programs in Scx-lineage cell subpopulations could identify dynamic changes in cellular phenotypes.

∗Pseudotime analysis is a computational method utilized in single-cell genomics to infer the temporal order of individual cells within a biological process or developmental trajectory (76). The method has several limitations such as the assumption of linear or simple topologies, the challenge of interpreting complex cellular dynamics and the frequent overestimation or underestimation of the underlying topology (77). For a comprehensive discussion of pseudotime analysis and its limitations, refer to relevant articles (77, 78).

#### Categorization of tendon cell subpopulations into functional groups

The integration of the different scRNAseq studies described above is challenging, since tendon cell subpopulations show not only diverse molecular markers but also highly specialized functions. Tendon cell subpopulations can be categorized into four major functional groups (Table 2):

1) Synthetic: This subpopulation is marked by the expression of genes such as *Col1a1* and *Fmod*, which specialize in producing and maintaining the ECM. These cells are involved in the early stages of tendon repair.
2) Regenerative: This subpopulation is involved in the later stages of repair. These cells contribute to ECM remodeling, the transition from inflammatory or fibrotic states to the remodeling phase, and the recovery of tendon structural and mechanical properties.
3) Inflammatory: Inflammatory subpopulations play key roles in initiating the tendon healing response, mobilizing other cells to clear damaged tissue and facilitating repair.
4) Fibrotic scar: The fibrotic subpopulation is associated with fibro-adipogenic progenitors. When the tendon is damaged, fibro-adipogenic progenitors are overactivated and produce an excessive amount of ECM components, which leads to scar formation.

**Table 2:** Subpopulation of tendon cells

Cells	Cluster name	Associated markers/genes	Phenotype/function	Ref.
Mouse tendon cells	Tendon fibroblasts 1	*Col1a1, Spp1*	Cell adhesion and migration	(68)
	Tendon fibroblasts 2	*Col1a1, Dpt*	Collagen fibril organization	
	Junctional fibroblasts	*Col1a1, Col22a1*	Maintenance of tendon structural integrity and functionality at junctional sites	
Human tendon cells	Tenocyte A	*Ptx3, Cxcl1, Cxcl6, Cxcl8*	Inflammatory response	(73)
	Tenocyte B	*Krt7, Scx, Fbn1, Mfap5, Vcan, Emilin1*	Production of tendon microfibrils	
	Tenocyte C	*Itga7, Tagln, Myl9, Acta2, Rgs5*	Formation of perivascular niche	
	Tenocyte D	*Apod, Col3a1, Cxcl14, Gsn, Lum, Dcn, Ly6e, Pdgfra*	Fibrotic response	
	Tenocyte E	*Tppp3, Prg4, Dcn, Clu, Lum, Prelp, Fmod, Comp, Crtac1, Cilp1/2*	Production of reparative matrix	
Rat tendon cells	Tendon fibroblasts 1	*Col1a1, Fmod, Comp, Chad, Sparc*	Maintenance of tendon core structure by producing type I collagen	(74)
	Tendon fibroblasts 2	*Apoe, Col3a1, Cfd, Tmsb4x, Gsn*	Production of circumferential collagen that wraps around the tendon	
Mouse injured tendon cells	Synthetic (C0)	*Tnmd, Col1a1, Fmod*	Tenogenesis and ECM organization	(75)
	Native tendon (C1)	*Coch, Chad, Car3*	Phenotypes that resemble uninjured tendon in structure and function	
	Reactive (C2)	*Mmp13, Lox, Fbln2*	ECM organization, collagen catabolic processes, and positive regulation of many cellular activities such as cell adhesion, migration, proliferation, collagen biosynthesis	
	Fibrotic (C3)	*Col3a1, Postn, Thbs3*	Fibrotic response through high levels of ECM synthesis	
	Inflammatory (C4)	*Saa3, S100a8, S100a9, Lcn2*	Inflammatory response	
	Muscle-associated (C5)	Not well-defined	Features of both muscle and native tendon	
Mouse tendon cells	Tppp3+ cells	*Tppp3, Pdgfra, Prg4*	Tendon regeneration	(63)
	T-FAPs (tendon fibro-adipogenic progenitors)	*Ly6a, Plin2, Pdgfra* (*Tppp3*-negative)	Fibrotic response	
	Tenocytes	*Fmod, Tnmd, Thbs4*	Maintenance of tendon structure and function	
Mouse tendon cells	Cluster I	*Nes, CD31, CD34*	Vascular or hematopoietic functions	(64)
	Cluster II	*Nes, CD146, Scx, Col1, Tnc, Mkx*	Contribution to development and repair	
	Cluster III	*Scx, Col1, Tnc, Thbs4, Mkx*	Maintenance of tendon structure and function	

*Acta2*, actin alpha 2; *Apod*, apolipoprotein D; *Apoe*, apolipoprotein E; *Car3*, carbonic anhydrase 3; *Cfd*, complement factor D; *Chad*, chondroadherin; *Cilp1/2*, cartilage intermediate layer protein 1/2; *Clu*, clusterin; *Coch*, coagulation factor C homolog; *Col1*, collagen type I; *Col1a1*, collagen type I alpha 1; *Col3a1*, collagen type III alpha 1; *Col22a1*, collagen type XXII alpha 1; *Comp*, cartilage oligomeric matrix protein; *Crtac1*, cartilage acidic protein 1; *Cxcl*, C-X-C motif chemokine ligand; *Dcn*, decorin; *Dpt*, dermatopontin; *Fbln2*, fibulin 2; *Fmod*, fibromodulin; *Fbn1*, fibrillin 1; *Gsn*, gelsolin; *Itga7*, integrin subunit alpha 7; *Krt7*, keratin 7; *Lcn2*, lipocalin 2; *Lox*, lysyl oxidase; *Lum*, lumican; *Ly6a*, lymphocyte antigen 6 family member A; *Ly6e*, lymphocyte antigen 6 family member E; *Mfap5*, microfibril associated protein 5; *Mkx*, mohawk homeobox; *Mmp13*, matrix metalloproteinase 13; *Myl9*, myosin light chain 9; *Nes*, nestin; *Pdgfra*, platelet-derived growth factor receptor alpha; *Plin2*, perilipin 2; *Postn*, periostin; *Prg4*, proteoglycan 4; *Prelp*, proline/arginine-rich end leucine-rich repeat protein; *Ptx3*, pentraxin 3; *Rgs5*, regulator of G-protein signaling 5; *S100a8*, S100 calcium binding protein A8; *S100a9*, S100 calcium binding protein A9; *Saa3*, serum amyloid A3; *Scx*, scleraxis; *Sparc*, secreted protein acidic and cysteine rich; *Spp1*, secreted phosphoprotein 1; *Tagln*, transgelin; *Thbs3*, thrombospondin 3; *Thbs4*, thrombospondin 4; *Tmsb4x*, thymosin beta 4 X-linked; *Tnc*, tenascin-C; *Tnmd*, tenomodulin; *Tppp3*, tubulin polymerization-promoting protein 3; *Vcan*, versican.

Understanding these functional groups could be useful for developing novel therapeutic strategies to enhance tendon healing, reduce fibrosis, and restore original tendon function.

### Strategy of mitigating fibrotic scar formation in tendon healing

#### Difference between adult and fetal/neonatal tendon healing

Since adult tendon injuries often heal with the formation of fibrotic scar, the idea of scarless healing is particularly attractive. While adult tendon injury heals slowly, fetal tendon injury heals rapidly without the formation of scar tissue (“scarless repair”; See Ref (79) and Review (80)).

Fetal wound healing is characterized by a markedly diminished or even absent inflammatory response. An *in vitro* study using the proinflammatory cytokine interleukin (IL)-1β at 100 pM for 24 h to mimic inflammatory conditions showed that postnatal mouse tendon cells respond to IL-1β treatment with significantly higher levels of inflammatory mediators, including IL-6, tumor necrosis factor-α (TNF-α), cyclooxygenase 2 (COX2), matrix metalloproteinase (MMP) 3, and MMP13, compared to embryonic mouse tendon cells (81). A study using a sheep model found that there is minimal expression of TGF-β in the fetal tendon wound, while the expression of TGF-β is significantly upregulated in the adult tendon wound (82). Thus, it is tempting to speculate that suppression of the inflammatory response to adult tendon wound healing by decreasing active TGF-β levels could result in reduced scar formation.

There is a notable study that compares the healing mechanisms between neonatal and adult tendons using a complete tendon transection model in mice (41). In neonatal tendon healing, αSMA-expressing cells are transiently recruited to the injury site. Thereafter, residential tenocytes migrate to the injury site and lead to tissue regeneration and restoration of function. However, this is not the case in adult tendon wound healing. In adult healing, although αSMA-expressing cells are initially recruited to the injury site, residential tenocytes do not migrate there, and the αSMA-expressing cells persist there. As a result, the αSMA-expressing cells form a permanent scar with impaired functional properties (41). This study indicates that intrinsic recruitment of Scx-lineage cells is a key cellular mechanism of neonatal tendon healing, but that this cellular mechanism is absent in adult tendon healing. Recently, it has been shown that TGF-β signaling is required for tenocyte recruitment in neonatal mouse tendon regeneration (44). Mimicking fetal and neonatal healing processes may be the key to success in the treatment of adult tendon injuries.

#### Potential strategy of mitigating fibrotic scar formation in tendon healing

As described above, TGF-β signaling is centrally positioned to be able to reduce scar formation in adult tendon healing because it is TGF-β itself that increases the number of myofibroblasts and sustains fibrotic scar formation (83). Indeed, a *Smad3* tendon-specific KO mouse shows reduced scar formation in a tendon transection model and a 42% lower tensile force in healing tendon (84). Therefore, even if challenging, it would be a more reasonable choice to inhibit a specific downstream effector in TGF-β signaling that regulates fibrotic scar formation, which could result in the maintenance of better mechanical properties in the healing tendon. For instance, plasminogen activator inhibitor-1 (PAI-1) is a downstream mediator in the TGF-β-induced fibrotic healing process (85). PAI-1 KO mice show reduced fibrotic scar formation in injured flexor tendons without loss of mechanical properties (86). This model offers the possibility of inhibiting fibrotic scar formation without diminishing the beneficial aspects of TGF-β in tendon healing.

It is likely that inflammation plays dual roles in tendon healing: one role is to promote regenerative processes, and the other is to promote fibrotic processes. An important issue for modifying the healing response to injury is how to enhance tendon regeneration without a concurrent increase in fibrotic scar formation. In this context, one question is when we should start antiinflammatory treatment during the healing process. For instance, a subcutaneous injection of the antiinflammatory drug dexamethasone during a period of 0 to 4 days post injury (the early inflammatory phase) in the rat Achilles tendon transection model significantly disimproves the mechanical properties of the healing tendon compared to the administration of saline (87). In contrast, the same treatment during a period of 7 to 11 days post injury (the early proliferative phase) significantly improves the mechanical properties in the rat healing tendon (88). Importantly, treatment with dexamethasone during a period of 7 to 11 days significantly upregulates Scx and downregulates αSMA protein levels in the rat healing tendon (89). Thus, we propose that antiinflammatory treatment in the early proliferative phase could promote tendon healing and prevent fibrotic scar formation with downregulation of myofibroblast activity.

## Topic 3: Mechanical loading in tendon

### Mechanotransduction in tendon cells

Growing evidence indicates that mechanical signals could serve as a trigger for biochemical signals, which then drive essential cellular processes such as differentiation, proliferation, tissue development, and maintenance (90, 91, 92). The mechanotransduction pathway includes interactions with primary cilia and integrin-containing focal adhesions, activation of cell-surface ion channels, changes in the levels of second messengers such as intracellular calcium ions or ATP, and rearrangements of the cytoskeleton (93).

Recent studies have made significant advances, opening new avenues in mechanotransduction processes, particularly in the signaling pathways of Yes-associated protein (YAP) and transcriptional coactivator with PDZ-binding motif (TAZ). YAP/TAZ molecules are central components of the Hippo signaling pathway and are known as mechanoresponsive transcription factors (94). Mechanical loading triggers the nuclear translocation and altered transcriptional activity of YAP/TAZ (95), which in turn regulate the expression of tenogenic markers such as *Col1*, *Scx*, and *Tnmd* in TSPCs (96). Reduced cellular tension rapidly decreases chromatin accessibility and induces matrix degradation through an epigenetic and transcriptional YAP/TAZ axis (97). It remains to be elucidated how the YAP/TAZ pathway regulates tendon mechanobiology, making this a significant area for future investigations. While mechanosensory molecules such as YAP/TAZ are thought to contribute to tendon wound healing, the following sections focus on how mechanical loading interacts with tendon-related transcription factors and TGF-β signaling.

### Effects of mechanical loading on tendon-related transcription factors and TGF-**β**

Mechanical loading plays a crucial role in upregulating tendon-related transcription factors such as Scx, Mkx, and EGR1. Among these factors, Scx responds robustly to mechanical loading such as treadmill running and dynamic stretching. A 6-week treadmill training program with physiological loading markedly elevates *Scx* gene expression in the epitenon fibroblasts of adult mice (98). These fibroblasts migrate from the epitenon into tendon fascicles, suggesting a direct link between mechanical loading and cellular mobilization in tendons. Similarly, cyclic loading has been shown to enhance the expression of *Scx* and *Col1a1* to a greater extent than static loading in 3D bioartificial tendons using a mouse multipotent mesenchymal cell line (C3H10T1/2), underlining the dynamic nature of the tendon response to physical stress (99). A study has been conducted using the plantaris tendon mechanical overload model in conditionally deleted *Scx* (*Rosa26*^*CreERT2/CreERT2*^ *Scx*^*fl/fl*^ mice [*Scx*^*Δ*^]) and their WT counterparts (*Scx*^*+*^) (100). In this study, *Scx*^*+*^ mice exhibited more pronounced tendon growth through differentiation of CD146+ pericytes into tenocytes compared to *Scx*^*Δ*^ mice (100), revealing the importance of Scx in the growth of adult tendons in response to mechanical loading.

Mkx, another transcription factor in tendons, also plays an important role in the mechanoresponsive pathway in tenogenesis. WT mice subjected to treadmill exercise show an increase in collagen fiber diameter and density in response to mechanical loading, while *Mkx*^*−/−*^ mice fail to respond to the same mechanical stimulation (101). Mechanical stretching in primary rat tenocytes results in the translocation of general transcription factor II-I repeat domain-containing protein 1 (*Gtf2ird1*) from the cytoplasm to the nucleus and activates the Mkx promoter (101). Under mechanical stretch stimulation *in vitro*, *Mkx*^−/−^ rat tendon-derived cells show chondrogenic differentiation, whereas WT rat tendon-derived cells show tenogenic differentiation (102). These findings indicate that Mkx is actively involved in the mechanoresponsive pathway during tendon differentiation.

EGR1 is known to respond to mechanical signals in tendon cells. In *in vitro* 3D-engineered tendons constructed with mouse MSCs (C3H10T1/2), the expression of *Egr1* is upregulated by mechanical loading and downregulated by its loss (103). In a mouse Achilles tendon injury model, reduced mechanical loading induced by the injection of botulinum toxin (botox) to cause muscle paralysis significantly decreases the expression of the *Egr1*, *Scx*, *Tnmd*, *Col1a1*, *Col1a2*, and *Tgfb2* genes (103). In 3D-engineered tendons and tendon healing models, interestingly, EGR1 overexpression in tenocytes rescues the downregulation of tendon-related genes such as *Egr1*, *Scx*, *Tnmd*, *Col1a1*, *Col1a2*, and *Tgfb2* even in the absence of mechanical loading (103). These observations suggest that EGR1 upregulates the transcriptional response to molecules which are responsible for mechanotransduction signaling pathways in tenocytes, and that EGR1 could promote tendon wound healing even in the absence of mechanical loading.

TGF-β is known to be activated in response to mechanical force, and it promotes tenocyte morphogenesis during tendon development (104). The activity of TGF-β/SMAD2/3 signaling is downregulated in chick limb tendons under immobilization by rigid muscle paralysis where muscle contraction is disrupted (30). Moreover, the treatment of limb explants with TGF-β2 leads to a higher expression of *Scx*, *Tnmd*, *Thbs2*, and *Smad7* (30). We have previously provided compelling evidence that mechanical force upregulates the expression of Scx in mouse tenocytes through the activation of the TGF-β/Smad2/3-mediated pathway (72); indeed, mechanical force at physiological levels maintains the expression of Scx. However, the sudden loss of mechanical force such as occurs in complete tendon transection in mice induces an excessive release of active TGF-β from the ECM and causes massive tenocyte death (72). Thus, this experimental evidence directly links an excessive release of active TGF-β from the ECM to adult tendon pathology, emphasizing the importance of mechanical force of an appropriate magnitude in maintaining tendon integrity by regulating biochemical signals that control cell viability and ECM composition. These findings highlight the pivotal role of mechanical force in sustaining tendon homeostasis through coordinated biochemical pathways and cellular responses.

### Synergistic effects of transcription factors and mechanical loading on tenogenic differentiation in stem/progenitor cells

Biomechanical stimuli could be applied to promote the differentiation of stem/progenitor cells toward tenogenic lineage cells during tendon healing. The overexpression of transcription factors such as *Scx* or *Mkx* in human or mouse MSCs has been clearly shown to effectively induce tenogenic differentiation *in vitro* (26, 28, 105), and an interesting observation is that this effect is synergistically enhanced in combination with mechanical loading. For instance, Scx overexpression boosts tenocyte maturation in human embryonic stem cell-derived MSCs when subjected to mechanical loading *in vitro* (106). This process occurs through antagonizing osteogenic differentiation mediated by BMP2-smad-Runx2 (106). In contrast, *Scx*-null mouse tendons undergo ectopic ossification in response to tendon injury (42). The combined application of Scx overexpression and stretching significantly increases the expression of tenogenic markers such as *Scx*, *Mkx*, *Bgn*, and *Thbs4*, and promotes new collagen deposition in human iPSC-derived MSCs *in vitro* (107). Immunostaining of type I collagen in iPSC-derived MSCs overexpressing *Scx* mechanically loaded for 7 days reveals a more organized collagen fibril network which is parallel to the actin fibers and perpendicular to the axis of stretch (107). Moreover, in another study using equine tenocyte-derived iPSCs, forced reprogramming of tenocytes by Yamanaka factors (Oct3/4, Sox2, Klf4, and c-Myc) repressed the expression of tenogenic genes, including *Egr1*, *Col1a2*, *Dcn*, and *Tnc*, in tenocyte-derived iPSCs (108). However, these repressed tenogenic genes could be re-activated by the overexpression of Mkx in combination with mechanical loading, and their expression levels were greater than those observed in mechanical loading alone or overexpression of Mkx alone *in vitro*. These investigations illuminate the complex processes by which mechanical loading and genetic factors synergistically direct stem/progenitor cells toward a tenogenic fate. The extent of mechanical loading and levels of transcription factor expression that are optimal for improving tendon healing remain to be elucidated.

### Optimal mechanical loading for promoting tenogenic responses *in vitro*

A complex issue is that the optimal percentage of mechanical loading for tendon healing depends on multiple factors. *In vitro* studies on mechanical loading are useful for dissecting the complex interplay between mechanical load and tendon cells (109). They provide insights into tenogenesis, tendon cell homeostasis, and tendon wound healing after an injury. For instance, uniaxial strain mimics the loading conditions in native tendon cells (109). In a 2D *in vitro* uniaxial loading model (Table 3), the optimization of both the mechanical strain and the loading parameters is important to mimic the physiological conditions that promote tendon repair and regeneration (109). A mechanical strain range of 4% to 8%, which mimics the physiological tendon strain, has been shown to be optimal for promoting tenogenic responses to tenocytes *in vitro* (110, 111, 112). Mechanical strain in this range increases the expression of tendon-specific markers, enhances collagen synthesis, and maintains cellular alignment (110, 111, 112). In contrast, a study of 2D *in vitro* uniaxial loading of human tenocytes at a lower strain level (3.5%) showed upregulation of inflammatory markers such as *Il1b* and *Cox2* and of the catabolic enzyme *Mmp3* (113). Interestingly, higher strain levels, above 8 to 10%, when applied to human tenocytes, replicate overuse injuries and induce catabolic effects, including tendon degeneration, inflammation, and increased expression of matrix metalloproteinases to degrade the ECM (114, 115). Mechanical loading of optimal magnitude and frequency promotes anabolic effects that aid tendon repair. In a study using 3D bioartificial tendons seeded with murine fibroblasts, physiological loading (4% elongation) promoted the expression of tenogenic genes such as *Scx*, *Mkx*, *Tnmd*, and *Col1a1*, whereas overload (8% elongation) increased the expression of *Mmp3* and the protein levels of IL-6, while decreasing the expression of tenogenic genes (116). In a study using 3D constructs with rabbit TSPCs in an underloading environment (3% cyclic tensile strain at 0.25 Hz for 8 h per day), TSPCs differentiated into osteogenic rather than tenogenic cells, leading to heterotopic ossification (117). These findings clearly highlight two important unresolved issues: (1) how to optimize the loading conditions to elicit beneficial cellular responses to mechanical loading and (2) how to avoid detrimental catabolic effects.

**Table 3:** Effect of mechanical strain on stem cells and tendon fibroblasts in 2D dynamic uniaxial loading conditions *in vitro*

Strain level	Frequency, duration	Cell	Outcomes		Effects	Ref.
			Tendon-related and others	ECMs		
2%	0.25 Hz, 6 h	Rat tenocytes	Increased gene expression of *Mkx*, *Tnmd*	Increased gene expression of *Col1a1*, *Col1a2*	Promote tenogenesis	(101)
3.5%	1.0 Hz, 2 h	Human flexor digitorum profundus tendon cells	Increased gene expression of *Il1β*, *Cox2*, *Mmp3*		Pro-inflammatory	(113)
5%	0.5 Hz, 24 h	Porcine patellar tendon fibroblasts		Increased gene expression of *Col1*, *Dcn*	Promote tenogenesis	(110)
6%	0.25 Hz, 8 h	Murine TSPCs	Increased gene expression of *Scx*, *Mkx*, *Tnmd*	Increased gene expression of *Col1a1*	Promote tenogenesis	(111)
4%	0.5 Hz, 4 h	Human patellar tendon fibroblasts	Decreased gene expression of *Cox2*, *Mmp1*		Antiinflammatory	(115)
			Decreased protein production of PGE_2_			
8%	0.5 Hz, 4 h	Human patellar tendon fibroblasts	Increased gene expression of *Cox2*, *Mmp1*		Proinflammatory	(115)
			Increased protein production of PGE_2_			
4%, 8%	0.5 Hz, 4 h	Human patellar tendon fibroblasts	Increased gene expression of *Tgfb1*	Increased gene expression of *Col1*	Promote tenogenesis	(112)
			Increased protein production of TGF-β1	Increased protein production of type I collagen		
8%, 12%	0.5 Hz, 24 h	Human patellar tendon fibroblasts	Increased protein production of PGE_2_, COX-1, COX-2		Proinflammatory	(114)

*Col1*, collagen type I; *Col1a1*, collagen type I alpha 1; *Col1a2*, collagen type I alpha 2; COX, cyclooxygenase; *Cox2*, cyclooxygenase 2; *Dcn*, decorin; *Il1β*, interleukin 1 beta; *Mkx*, mohawk homeobox; *Mmp1*, matrix metallopeptidase 1; *Mmp3*, matrix metallopeptidase 3; PGE_2,_ prostaglandin E2; *Scx*, scleraxis; *Tgfb1*, transforming growth factor beta 1; *Tnmd*, tenomodulin; TSPCs, tendon stem progenitor cells.

### Optimal mechanical loading for tendon wound healing in animal models

Several animal models have been employed to investigate the effects of mechanical loading on tendon healing. For example, in a rat Achilles tendon transection model, 5 min of treadmill walking for 4 days significantly improve the mechanical properties of the healing tendon (118). Another study examined rat transected Achilles tendon wound healing under three different loading conditions, (1) no loading (calf muscle paralysis through botox injections, combined with joint fixation using a steel orthosis); (2) mild loading (botox injections alone); and (3) strong loading (free cage activity) (119, 120). Both the mild and strong loading groups showed increased expression of collagen and other ECM genes and improved mechanical properties of the healing tendon. A negative result in the strong loading group was that only this group showed increased inflammatory gene expression and microdamage. Thus, the take-home message is that mild loading is sufficient to improve the quality of healing tendon without an inflammatory response or structural damage.

### Clinical relevance

In the clinic, traditionally, ankle immobilization without weight bearing was the most common rehabilitation regimen that is used for both surgical and nonsurgical treatments in human acute Achilles tendon rupture (121, 122). Ankle immobilization with plaster casts stabilizes the ruptured tendon ends during the healing process, but it increases the risk of complications, including ankle joint stiffness and gait abnormalities (123, 124). A modern “functional brace” is a type of orthopedic device designed to provide both stabilization and movement in a specific joint or body part. Recent studies have shown that early ankle motion, starting within 2 weeks post operation and using a functional brace, improves joint mobility without causing tendon elongation (122, 125). However, the translation of optimal loading conditions from the experimental studies described above into evidence-based protocols for promoting tendon healing remains an unmet need. A study investigated the effects of early progressive tensile loading in patients following surgical repair of Achilles tendon rupture (126). A tensile loading of 30 N twice daily was initially applied for 2 weeks post operation, and then the loading was increased step by step up to 225 N. The outcome showed a higher elastic modulus in the healed tendons, along with improved mechanical properties, suggesting that early tensile loading has clear benefits for improving the mechanical properties of the healing tendon. These findings highlight the importance of early mobilization and controlled tensile loading in promoting tendon healing, which could support the development of advanced rehabilitation protocols for improved recovery outcomes.

## Perspectives

Although adult tendon wound healing is a complex process, we urgently need to bridge the gap to clinical challenges. It has been well-documented that Scx-expressing TSPCs contribute to tendon wound healing. Recently established analytical systems such as lineage cell tracing and single-cell analysis are promising and offer a comprehensive view of cellular behavior, including functional diversity, spatial distribution, and temporal dynamics. In this context, they are highly attractive and useful for further detailed functional analyses. However, the current problem is that we still do not thoroughly understand the basic cellular and molecular mechanisms of tendon wound healing following injury. It is unknown when and how each progenitor cell subpopulation is triggered for differentiation into mature tenocytes to participate in the healing process. We need to clarify the extent to which each progenitor cell subpopulation is involved in fibrotic healing following injury, and which subpopulation(s) contribute to and drive fibrotic scar formation. The critical factor(s) and/or molecule(s) by which fibrotic tendon healing changes to (abnormal) scar formation also remain to be elucidated.

**Figure 3:**
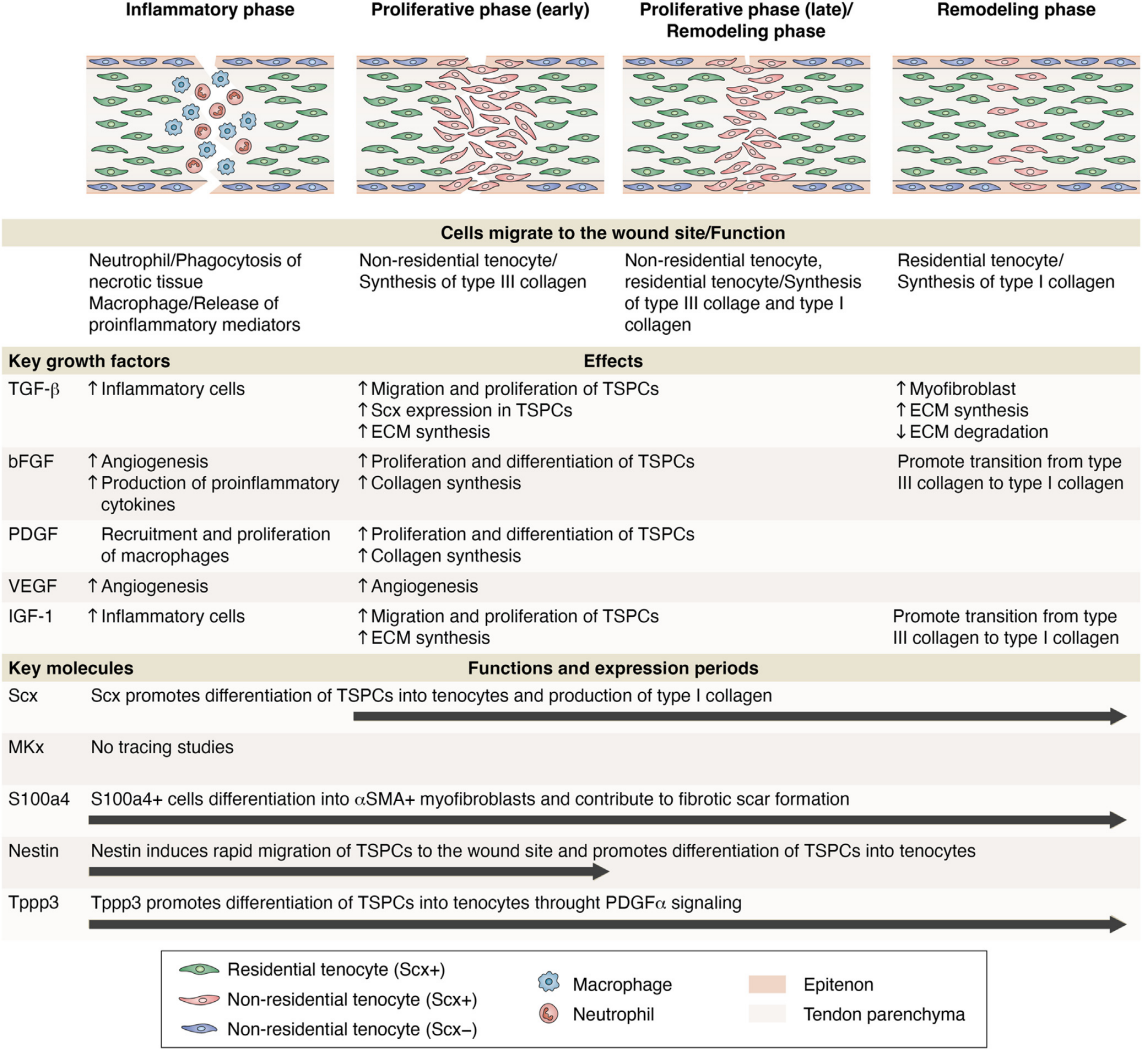
**Schematicillustration ofcellularcontributions, key growth factors, and key molecules involved in each phase of healing following tendon injury.**

Here, we would like to argue for a conceptual model of adult tendon healing (Fig. 3), and we present the following scenario with proposed factors/molecules for appropriate tendon wound healing:

(1) Which cell type(s) should initially migrate to the wound site?

Since residential tenocytes are delayed in migrating to the wound site, nonresidential TSPCs need to be rapidly recruited from outside the tendon parenchyma (peritenon or tendon sheath). Sca-1+ cells, nestin+ cells, and Tppp3+ cells are potential candidates for this role in the early proliferative phase. These cells have different characteristics: nestin+ cells express Scx before injury, start to migrate to the wound site during the inflammatory phase and persist for a short duration, peaking at around 1 week; Tppp3+ cells are induced to express Scx and begin migrating to the wound site during the inflammatory phase, persisting for an extended period (from 1 to at least 4 weeks); and Sca-1+ cells are induced to express Scx and gradually migrate to the wound site during the proliferative phase.

(2) Interactions between αSMA-expressing myofibroblasts and Scx-expressing cells from TSPC subpopulations

Since αSMA-expressing myofibroblasts and Scx-expressing cells are documented to play indispensable roles in tendon wound healing, their optimal proportion should be critical in each healing phase. Up to the late proliferative phase, αSMA-expressing myofibroblasts play a role in the initial organization of the ECM. It remains to be elucidated how heterogenous populations of “myofibroblast subtypes” can affect the deposition and/or reorganization of the ECM at the wound site. In the late proliferative-remodeling phases, Scx-expressing cells should be augmented to remodel the ECM, which is initially organized by αSMA-expressing myofibroblasts. This is supported by the fact that the inactivation of the *Scx* gene during tendon wound healing results in incomplete remodeling of type III to type I collagen (42). Additionally, a reduction in the proportion of αSMA-expressing myofibroblasts could improve ECM remodeling and prevent fibrotic scar formation (*e.g.*, the S100a4 haploinsufficiency model). The current unresolved question is what kind(s) of interrelationships exist between Scx-expressing cells and αSMA-expressing myofibroblasts during tendon wound healing, *i.e.*, could Scx-expressing cells negatively regulate αSMA-expressing myofibroblasts?

(3) Generation of essential cytokines/growth factor components

In the proliferative phase, one critical issue is to promote the proliferation, migration, and tenogenic differentiation of TSPCs. TGF-β can strongly promote these processes. In the early remodeling phase, TGF-β activity could support ECM reorganization and collagen maturation. In the late remodeling phase, TGF-β activity should be gradually reduced by downstream effectors such as PAI-1 to prevent fibrous scar formation. The growth factors PDGF and bFGF are also essential for promoting the proliferation and differentiation of TSPCs and collagen synthesis. Single or combined application of PDGF and bFGF could significantly enhance the healing process.

(4) Optimal mechanical loading and clinical application

The goal is to minimize the risk of reinjury and fibrotic scar formation. An appropriate mechanical loading is indispensable to promote tendon healing and to recover functional and mechanical properties in the healing tendon. As indicated in *in vitro* studies, mechanical loading at strain levels between 4% and 8% is optimal for tendon wound healing. From a clinical perspective, understanding the cellular response to mechanical loading can inform the development of more effective treatment methods and rehabilitation protocols for tendon injuries.

## Conflict of interest

The authors declare that they have no conflicts of interest with the contents of this article.
